# Guideline use among different healthcare professionals in diagnosing attention deficit hyperactivity disorder in Dutch children; who cares?

**DOI:** 10.1186/s40359-019-0304-1

**Published:** 2019-07-03

**Authors:** Birgit Levelink, Lonneke Walraven, Edward Dompeling, Frans J. M. Feron, Dorothea M. C. B. van Zeben-van der Aa

**Affiliations:** 1 Department of Paediatrics, Maastricht University Medical Centre (MUMC+), PO Box 5800, 6202 AZ Maastricht, The Netherlands; 2 Department of Social Medicine, Care and Public Health Institute (CAPHRI), Maastricht University, Maastricht, The Netherlands

**Keywords:** ADHD, Guidelines, Diagnosis, Children

## Abstract

**Objective:**

Current data about Attention Deficit Hyperactivity Deficiency (ADHD) guideline use in the Netherlands are absent. This study analysed ADHD guideline use among different healthcare workers, and the use of key elements from these guidelines to diagnose ADHD.

**Method:**

A survey assessing ADHD guideline use was distributed throughout the Netherlands to various health care professionals. Only professionals involved during the diagnostic process were included.

**Results:**

Response rate among GPs was low (111/1450), but high among other health care professionals (251/287). A total of 362 surveys were analysed, 186 responders (51%) were involved during the diagnostic process. Overall guideline use was 64.5%; the national multidisciplinary guideline or a guideline made by a professional’s own institution were most used. Psychiatrists, psychologists and paediatricians reported compliance with key elements of the guidelines such as gathering information from a third party (> 90%) and carrying out a developmental history (> 88%). Use of a standardized interview (< 52% often use) was low. Only paediatricians performed a physical examination regularly (88%).

**Conclusion:**

Despite low general use of guidelines, psychiatrists, psychologists and paediatricians use similar key elements of ADHD guidelines. This study provides opportunities to improve care through increasing familiarity with ADHD guidelines and the use of standardized interviews.

## Introduction

Attention-Deficit Hyperactive Disorder (ADHD) is a neurodevelopmental disorder, affecting people of all ages with an onset in childhood. According to the most recent meta-analyses, worldwide prevalence in children and adolescents is estimated between 3.4 and 7.2% [28, 29, 39]. Although no increase in the worldwide prevalence of ADHD was found in the past ten years, in 2014 the need for (health) care in relation to symptoms of attention deficit and/or hyperactivity and impulsivity in the Netherlands showed a 30 % increase over the previous decade [11, 15, 16]. Recent studies provided several explanations, such as increased awareness of ADHD among health care professionals, parents and teachers, increased academic research on the disorder, and better accessibility to (health) care [5, 14, 16, 32]. Little is known about the care pathways of Dutch children with problems of attention deficit and/or hyperactivity, and the use of, and compliance with ADHD guidelines by different healthcare professionals. It is important to know how an ADHD diagnosis ADHD is made, as deviation from recommendations may lead to undetected comorbid conditions, misdiagnosis and unnecessary use of tests. ADHD is a complex diagnosis in which both genetic and environmental factors play an important role [37]. During the diagnostic process it is important to assess that, due to symptoms of attention deficit and / or hyperactivity and impulsivity, the child experiences limitations in his or her functioning [3]. Correct interpretation of behavioral characteristics requires sufficient knowledge about the disorder. Worldwide research between 1995 and 2016 showed a lack of knowledge about ADHD and a shortage of enthusiasm among GPs to get involved in ADHD care. [10, 18, 20, 21, 33, 34, 36]. Epstein et al. [9] concluded that part of community-based American paediatricians did not act according to evidence-based guidelines [9]. As in many other countries, ADHD is diagnosed in the Netherlands by various healthcare professionals, such as general practitioners (GPs), psychologists, child & youth psychiatrists and pediatricians [6, 35, 38, 42]. Over time, several Dutch ADHD guidelines were published to standardize and improve diagnosis and treatment. In 2005 a multidisciplinary guideline was published which has many similarities with the guideline commissioned by the British National Institute for Health and Clinical Excellence [22, 26]. Since 2014 guidelines for GPs, youth care and primary youth health care have been issued, which gave the opportunity to diagnose and treat symptoms of ADHD in consultation with specialists [4, 40].

To gain more insight into the increase of (health) care use in the Netherlands due to ADHD symptoms, it is important to know whether important elements of an ADHD diagnosis are sufficiently taken into account by (health) care professionals to avoid misdiagnosis. For example, consideration should be given to the presence of ADHD behavior in different settings. In addition, it is useful to know if problems are adequately detected in primary health care. The first step is to gain insight into the use of the existing guidelines by different (health) care professionals, and to evaluate if these guidelines are applied correctly. The objective of this study was therefore 1) to describe the use of different ADHD guidelines among healthcare professionals for children in the Netherlands and 2) to determine whether diagnosing ADHD is in accordance with key elements of these guidelines.

## Methods

### Study design and setting

This cross-sectional study was conducted between March 2017 and August 2017 at the paediatric department of the Maastricht University Medical Centre. A survey was designed using the online questionnaire platform Qualtrics [30]. A wide variety of healthcare professionals may be involved in ADHD care, but exact data was not available. Therefore, all known institutions for ADHD care in the Netherlands were identified through searches on the Internet, to then evaluate whether they were involved in ADHD diagnostics. The targeted professional groups included paediatricians, child and youth psychiatrists, psychologists, GPs, general practice-based nurse specialists and youth health care physicians (school doctors). The Dutch ADHD Network distributed the survey directly to health care professionals affiliated with the network. In total 36 psychologists, 27 child and youth psychiatrists, 48 paediatricians and 13 youth health care physicians were directly addressed by the ADHD network, and 128 institutions for child mental health, 10 hospitals and 25 primary health services were approached. Among individual general practitioners it was difficult to determine if ADHD care was provided. GPs are organized per province in groups, and email addresses for individual GPs were requested from the presidents of these GP groups. Two provinces released this data, and therefore a sample of 1450 GPs was addressed.

### Measurement

A 27-question survey based on the different Dutch guidelines was developed, focusing on elements used during the diagnostic process. The first part of the survey consisted of 4 questions assessing involvement of the individual healthcare professional during the diagnostic phase of ADHD. Respondents not belonging to the target group were excluded after finishing this part. The following 16 questions related to the use of guidelines in general and evaluated the practice patterns with regard to an ADHD diagnosis. Survey items assessed adherence to five diagnostic key elements specified in the Dutch multidisciplinary guideline, the Dutch guideline for GPs and the youth healthcare guideline (Table 1).

**Table 1:** Dutch guideline recommendations; diagnostic key elements from the guidelines that are asked for during the survey

Evaluation of attention deficit, hyperactivity and impulsivity symptoms. Advised by all guidelines. Specifically asked were the use of semi-structured interviews, options: semi-structured interview with parents (Anxiety Disorders in Interview Schedule for DSM-IV (ADIS), Children's Aggression Scale (CAS), Kiddie Schedule for Affective Disorders and Schizophrenia for school aged children (K-SADS), Kiddie Disruptive Behavior Disorders Schedule (K-DBDS), Parental Account of Children's Symptoms (PACS), Parent Interview for Child Symptoms (PICS-4-dutch version), Semi-structured Clinical Interview for Children and Adolescents (SCICA)).	
Gathering information from somebody else than the parents and/or child. Advised by all guidelines. Specifically asked how information is gathered; questionnaires, semi-structured interview or direct observation.	
Use of questionnaires. Advised by the Dutch multidisciplinary guideline: Child Behavior Checklist (CBCL), Youth Self Report (YSR) and Teacher report Form (TRF). Advised by youth health care: Strengths and Difficulties Questionnaire (SDQ), ADHD Questionnaire (ADHD vragenlijst AVL). Other possibilities; CRS, Conner’s Rating Scale, Questionnaire for behavioural problems in children (Vragenlijst voor Gedragsproblemen bij Kinderen, VvGK)	
Knowledge of developmental history, family history and physical condition. Advised by all guidelines.	
Additional examination only advised on indication. Only advised on *indication* by all guidelines. Specifically asked: Complete neuropsychological testing, Intelligent Quotient test (IQ), didactic test, Electrocardiogram (ECG), laboratory tests.	

Questions about diagnostic instruments and (re) screening too1ls were tailored to the Dutch situation. Questionnaires advised by different guidelines and askes for in the survey were: 1. ‘ADHD Vragenlijst’ [AVL, The Dutch ADHD Questionnaire], a Dutch behavioural questionnaire for children aged 4 to 18 years that is based on the Conners’ Rating Scale for ADHD [41]. 2. The Child Behavior Checklist (CBCL) and the Teacher’s Report Form (TRF), both components of the Achenbach System of Empirically Based Assessment (ASEBA) [1] (Achenbach, 1991). 3.The Strengths and Difficulties Questionnaire (SDQ), a brief behavioural screening instrument [13]. Use of The Conners’ Rating Scale (CRS), a behavioural questionnaire designed to assess symptoms of ADHD, originally developed by C. Keith Conners in 1969 and revised in 1997, was added as an extra option. To gather the intended information multiple choice questions and 3-point and 5-point Likert-type scale measured responses were mainly used. For some questions an open text field was included automatically when the answer “otherwise” was chosen. A paediatrician, a research worker and GP from the University of Maastricht were asked to pilot test the questionnaire, whereupon its applicability was improved for primary as well as secondary health care professionals.

The last 7 questions assessed the characteristics of the health care professionals, like gender, age, work experience and experience with diagnosing ADHD in children. A question about the location of the institution was included to determine geographic diversity.

### Analysis

Responses were converted to IBM SPSS Statistics version 22 for Mac for further analysis [19]. First, univariate descriptive statistics were used to assess frequencies of responses by demographic variables. To determine guideline use in general and per profession Pearson chi square tests was used. This test was also applied to analyse the use of important guideline elements. For each guideline component, reported response ‘often’ or ‘always’ was contrasted with reported response ‘never’, ‘rarely’ or ‘sometimes’. Because numbers of respondents per professional were low, and varied between subgroups, subgroup analyses were not possible. Finally, univariate logistic regression was used to analyse if the use of a standardised approved national guideline led to more adherence to key elements of the guidelines than the use of a protocol of the own institution or any other protocol. Professionals who responded to having used the approved Dutch multidisciplinary guideline, the GP guideline or the youth health care guideline were marked as using a standardised guideline. They were compared with professionals who responded to having used a protocol from their own institute or a protocol made by themselves.

## Results

### Characteristics of respondents

**Fig. 1:**
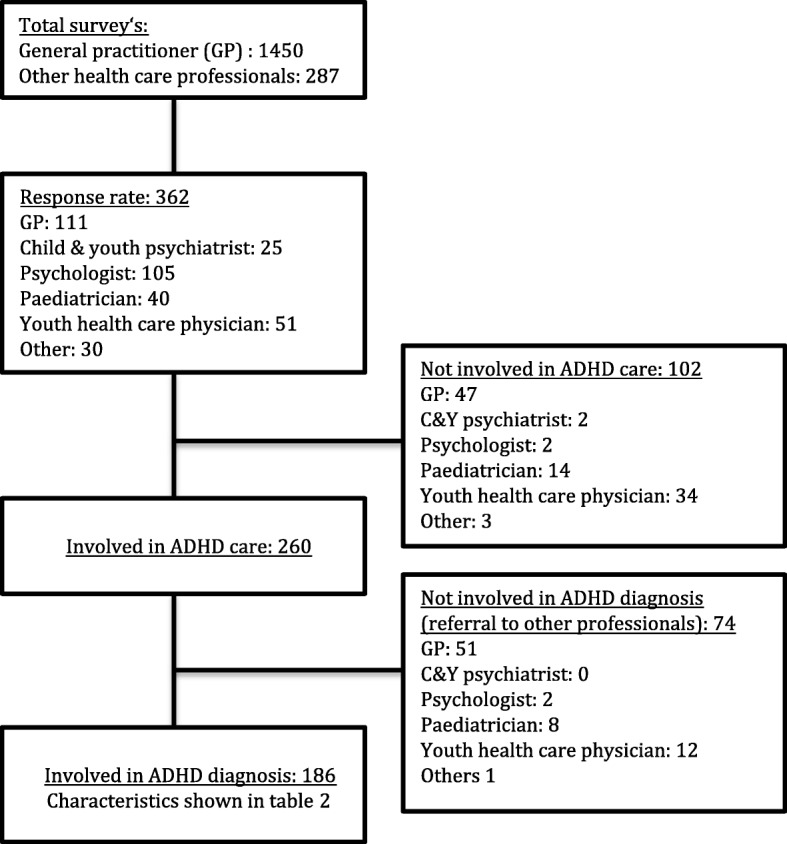
Response rate and included professionals

Response rate of GPs was low; 111 of the 1450 surveys returned. In contrast, the response rate of other health care professionals was high; 251 of 287 surveys were returned. In total 362 questionnaires were returned (Fig. 1). Only 186 professionals responded that they were actually involved during the diagnostic process of ADHD, 176 professionals referred children with symptoms of ADHD to another care professional. In particular youth health care physicians (45/50), GPs (98/111) and paediatricians (22/40) were excluded in the first part of the survey because they referred children when they suspected ADHD, and evaluated that additional diagnostics were necessary. 166 professionals completed the whole survey. Characteristics of professionals involved during the diagnostic process are shown in Table 2. The majority was female (84.3%). Most of the respondents evaluated less than 25 new patients per year (57.8%); especially pediatricians indicated that they had more than 100 consultations per year because of problems related to ADHD (41.2%). With the exception of GPs, respondents were equally distributed over the Netherlands.

**Table 2:** Characteristics of health care professionals involved during the diagnostic process

	Number of respondents N (%)
Profession (*n* = 186)	
Child & youth psychiatrist	23 (12)
Psychologist	101 (54)
Paediatrician	18 (10)
General practitioner	13 (7)
Youth health care physician	5 (3)
Remedial teacher	17 (9)
Other	9 (5)
Sex (*n* = 166)	
Female	140 (84)
Age in years (*n* = 166)	
20–35	65 (39)
36–55	74 (45)
56>	27 (16)
Number of new patients per year (*n* = 166)	
0–25	96 (58)
25–100	53 (32)
> 100	17 (10)

### Guideline use

The majority of professionals used some kind of guideline (64.4%). Of respondents who indicated that they did not use a guideline, 18.3% used their own protocol, and 17.2% used no protocol at all. Table 3 shows the use of guidelines by the four major response groups, i.e. child & youth psychiatrists, psychologists, paediatricians and GPs and the use of key elements from these guidelines. Psychiatrists used guidelines most frequently (81.8%). Standardized interviews, recommended in the official guidelines, were used by less than 52% of all professionals. Most used interviews were the Anxiety Disorders in Interview Schedule for DSM-IV (ADIS) and the Parent Interview for Child Symptoms (PICS-4-NL). Gathering information from a third party (e.g. school) was done by all disciplines. Information from a third party was gathered by observation (40%), standardized interviews (23%) and by questionnaires (34%). Although only recommended on indication in all guidelines, the majority of respondents performed an IQ test (70.5%). Only paediatricians performed physical examination regularly (88.3%). Comparison of the group using an approved standardized Dutch guideline with the group using a protocol from the own institute or made by the (health) care professional showed two significant differences (Table 4). Professionals who used an official Dutch guideline more often used a semi-structured interview (OR 2.1; 95% CI 1.1–3.7), and they were more likely to perform a physical examination (OR 2.6; 95% CI 1.1–5.9).

**Table 3:** Use of Dutch guidelines and diagnostic key elements from guidelines per healthcare professional

Dutch diagnostic guideline recommendations	Overall adherence (%; *N* = 169)	C&Y psychiatrists (%; *N* = 19)	Psychologists (%; *N* = 93)	Paediatricians (%; *N* = 17)	GPs (%; *N* = 13)	*p* value
General Guideline use	64.4	81.8	60.8	73.7	50.0	0.05
Guidelines used:						
Multidisciplinary guideline	38.6	72.2	33.3	50.0	14.3	
Youth healthcare guideline	11.4	0	14.0	14.3	0	
GP guideline	6.1	0	0	0	71.4	
Protocol own institution	39.5	27.8	49.1	21.4	0	
Not specified			3.6	14.3	14.3	
Evaluation of ADHD symptoms^a^						
Use of semi structured interview	51.7	68.2	49.5	63.2	50.0	0.09
Gathering information from third party^a^	92,8	94.7	97.8	94.1	50.0	< 0.001
Use of questionnaires^a^						
CBCL^c^	40.8	63.2	39.8	41.7		0.001 0.04
TRF^c^	53.8	73.7	59.1	64.7		
SDQ^a^	49,1	42,1	51.6	41.7	11.8	
AVL^a^	66.3	73.7	71.0	70.6	23.1	
CR-scale^c^	5.3	21.1	4.3	0		
Additional knowledge^a^						
Developmental history	89.2	100	96.7	88.2	16.7	< 0.001 < 0.001
Perform physical examination	19.2	10.5	7.6	88.3	25,0	
Additional examination used^b^						
neuropsychological tests	62.7	73.3	56.5	70.6	91.7	0.25 < 0.001
IQ tests	29,5	10,5	19.6	41.2	100	
Electrocardiogram	100	100	100	100	100	
Laboratory tests	100	100	100	100	100	

^a^use often or always

^b^use never or rarely on occasion

^c^Use of this instrument was not asked in version of questionnaire for GPs and youth health care physicians

*C&Y psychiatrist* Child and Youth psychiatrist, *GPs* General Practitioners, *AVL* ADHD Vragenlijst (Dutch ADHD Questionnaire), *CBCL* Child Behavior Checklist, *TRF* Teacher’s Report Form, *SDQ* Strengths and Difficulties Questionnaire, *CRS* Connor’s Rating Scale, *IQ* Intelligence Quotient

**Table 4:** Likelihood of using key elements when using a Dutch national approved ADHD guideline (instead of a protocol made by the own institution or a protocol made by the healthcare professional)

	Odds ratio	95% CI
Use of semi structured interview	2.1	1.1–3.7
Gathering information from a third party	1.7	0.5–5.7
Use of questionnaires		
CBCL	0.9	0.5–1.8
SDQ	1.6	0.9–2.9
AVL	0.6	0.3–1.2
Use of developmental history	1.6	0.6–4.2
Perform physical examination	2.6	1.1–5.9
Use of additional examination		
Neuropsychological tests	0.8	0.5–1.6
IQ tests	0.7	0.4–1.5

*CI* Confidence Interval, *CBCL* Child Behavior Checklist, *SDQ* Strengths and Difficulties Questionnaire, *AVL* ADHD Vragenlijst (Dutch ADHD Questionnaire), *IQ* Intelligence Quotient

## Discussion

As data about ADHD guideline use in the Netherlands was lacking, this study analysed ADHD guideline use among different healthcare workers, and the use of key elements from these guidelines to diagnose ADHD. The use of national approved Dutch guidelines was low, but was in accordance with the results of studies in other countries [7, 8, 31, 44]. Many of the responding professionals commented to have a protocol of their own institute based on the national guidelines. These institution protocols probably have many similarities with the national approved guidelines; the overall use of important diagnostic key elements, like gathering information from a third party and performing a developmental history was high, both in accordance with the different national guidelines. The only significant difference between the group using an approved national guideline and the group using an institution protocol was the use of a semi-structured interview and performing a physical examination. The more positive response on the question concerning physical examination in the group using a national approved guideline was not simply explained by the use of this approved guideline. Paediatricians were the only professionals who responded to perform regular physical examinations. All other professionals hardly used a physical examination as part of the diagnostic process. Physical problems, like visual and hearing impairment may mimic ADHD, and ADHD can also be part of a physical disease like neurofibromatosis. Children with ADHD often have somatic comorbidities like enuresis, making physical examination an important part of the diagnostic process [2, 12, 17, 25]. It seems necessary to reaffirm the importance of the physical examination to several professionals.

There were some striking features. Overall use of semi-structured interviews was low. ADHD is a best practice diagnosis, but diagnostic clinical structured interviews showed high values for sensitivity and specificity in relation to the comprehensive best practice diagnosis [27]. Low use of structured interviews may either lead to inaccurate diagnosis or undetected comorbidities. The heterogeneity of obtaining information regarding symptoms of inattention and hyperactivity from third parties and the high use of IQ tests and neuropsychological tests, by psychologists, C&Y psychiatrists and paediatricians was also remarkable. It is possible that our respondents evaluated a selected patient population with high comorbidity rates, requiring a tailored child-focused program, with more need for additional tests. However, part of these additional IQ and neurological tests may have been unnecessary, and therefore increased costs for ADHD care.

Currently various healthcare professionals in the Netherlands diagnose ADHD. Despite the introduction of several ADHD guidelines for primary healthcare workers, the majority of GPs and youth health care workers indicated to refer children with ADHD symptoms to other professionals. The National Health Statistics Report of the United States of America showed a high involvement of paediatricians; in their study almost 40% of the parents were told by a paediatrician that their child had ADHD [23, 42]. Many paediatricians in the Netherlands indicated not to be involved when it comes to diagnosing ADHD. Mental health care workers, in particular child psychologists and psychiatrists, were most involved during the diagnostic process. This study was conducted after the transformation of youth care in 2015 in The Netherlands, which might explain low involvement of paediatricians [24]. The transformation changed the financing system; municipalities instead of healthcare insurances became responsible for ADHD care both in terms of contents and finance. After the transformation, only hospitals with an arrangement with the municipalities were allowed to deliver ADHD care, and many paediatricians decided not to provide care to children with problems related to ADHD any longer. In this study, 50% of the responding paediatricians indicated themselves to be specialized in ADHD care, which corresponded with the large amount of children they said to evaluate every year. An explanation for this could be that municipalities particularly contracted paediatricians with large practices after the transformation of youth care. This could also explain the relatively high use of additional tests; these large practices are often highly specialized and therefore see children with complex problems.

This transformation of youth care was part of the new Child and Youth Act, which formed the basics of a plan of action made by all professionals involved in the care for children with (symptoms of) ADHD in 2015 [24]. The three major principles of this Child and Youth Act were: to make more use of ‘own strength’ and the social network of children and their parents; to allow children to participate as much as possible by normalizing, unburdening and not unnecessarily medicalize, and: to reduce specialised health care by using more primary care [43]. The number of included GPs in the study was low, maybe because they were only involved in ADHD care for children since 2014. Most GPs referred children directly after presenting with symptoms of ADHD or when they suspected co-morbidity. Due to the low number of GPs, no conclusions could be drawn for this group. It is important to do more research on the involvement of GPs in the care for children with ADHD, as making use of primary care professionals is an important principle of the new Child and Youth Act.

This study has several limitations. It was impossible to include professionals randomly by inviting the targeted group, because no exact data of professionals involved in ADHD care in the Netherlands was available. This has created various risks for selection bias. First, it is not clear whether a good reflection of care providers has received the questionnaire. Second, respondent bias was possible due to self-selection of the respondents; most likely professionals who felt involved in ADHD care completed the survey. Another limitation was the low number of some professionals. Response rate in the GP group was extremely low and the number of GPs involved in ADHD care even lower. As a result, no conclusions could be made concerning ADHD care by GPs. As opposed to all other professionals who were located all over the Netherlands, GPs from only two provinces could be approached. These provinces were representative for a part of the Netherlands, but not for provinces were the major cities are located. Finally the survey focussed on the diagnostic process and did not include treatment (both pharmacological and non pharmacological). It was a deliberate choice to exclude professionals who were not involved in the diagnostic process at the beginning of the survey. This was to prevent people who were not involved in ADHD diagnostics from completing the questionnaire and thus influencing the results negatively. Retrospectively, it would have been interesting to know whether the group that was excluded at the beginning of the questionnaire was involved in the treatment of ADHD. According to the guidelines, ADHD symptoms must be regularly evaluated during treatment, to determine to what extent ADHD symptoms still lead to dysfunction. In order to evaluate ADHD symptoms properly, sufficient knowledge about the disorder is essential; the question is whether this knowledge is sufficient if you do not participate in diagnosing ADHD. Further research is necessary to gain insight in the knowledge of professionals who only treat children with ADHD.

### Clinical implications

ADHD is a best-practice diagnosis. This was a quantitative study and no statements can be made about the quality of the ADHD diagnosis made by individual health care professionals or the possible impact on the increased demand for ADHD care. However, practice variations were identified which generated new hypotheses. Involvement of (relatively cheap) primary care was low. Response rate from the GPs was very low. The Dutch government wants a prominent role for GPs in the diagnostic process of ADHD so it is important to conduct more research on the involvement and knowledge of ADHD in this group.

Use of (expensive) additional testing was high, which may be linked to easy access to these resources, different demands of the referred patient group or low use of nationwide guidelines. Implications of these patterns cannot be derived from this study but are of interest for further investigation, especially directed to proper use of additional (neuro) psychological testing. Finally, more attention should be paid to the use of standardized interviews, for example by incorporating them in new guidelines.

## Conclusions

Various health care professionals, working in primary, secondary and tertiary care, diagnose ADHD in children in the Netherlands differently. In particular mental health care workers and specialized pediatricians are involved in the diagnostic process. A slight majority is using a nationwide guideline or a protocol of the professional’s own institution based on national approved guidelines. Adherence to guidelines differs per health care profession, but the use of diagnostic key elements, like use of information from a third party and a developmental history, is high among professionals with the highest response rate. Use of semi-structured interviews and physical examination is low, raising opportunities for improvement.
